# The relationship between fathers’ involvement in infant care and infant attachment levels

**DOI:** 10.1007/s11845-026-04281-7

**Published:** 2026-02-28

**Authors:** Kamile Akça, Suat Tuncay

**Affiliations:** 1 Faculty of Health Sciences, Gaziantep Islam Science and Technology University, Gaziantep, Türkiye; 2 Faculty of Health Sciences, Bingöl University, Bingöl, Türkiye

**Keywords:** Attachment, Fathers, Infant, Infant care, Paternal

## Abstract

**Background:**

Increasing recognition of the father’s role in early childhood development has led to growing interest in how father involvement in infant care influences the quality of paternal-infant attachment.

**Aims:**

This study aimed to determine the association between fathers’ involvement in infant care and paternal-infant attachment levels.

**Methods:**

This descriptive study involved 255 fathers, utilizing the “Descriptive Information Form,” the “Paternal-Infant Attachment Scale,” and the “Fathers’ Involvement in Infant Care Questionnaire” for data collection. Data analysis included the use of number and percentage distributions, mean and standard deviation calculations, t-test, one-way analysis of variance (ANOVA), and post hoc analysis. Additionally, Pearson correlation and linear regression analyses were conducted for a comprehensive examination of the obtained data. Institutional permission and ethics committee approval were obtained to conduct the study.

**Results:**

In this study, elevated levels of father and mother education, a consistent income, shorter marital duration, excellent spousal relationship status, fewer children, and increased time spent with infants were associated with heightened father involvement in infant care and higher paternal-infant attachment scores. A positive correlation was observed between fathers’ involvement in infant care and paternal-infant attachment. Furthermore, regression analysis results show that as fathers’ involvement in infant care increases, their attachment with their infant also strengthens.

**Conclusions:**

This study identified a positive correlation between fathers’ engagement in infant care and the level of attachment between fathers and infants. To strengthen the paternal-infant attachment, fathers should be encouraged to involvement in infant care.

## Introduction

During the initial years of life, newborns rely on caregivers to fulfill their fundamental requirements for survival and development [1]. Typically, mothers assume the primary responsibility for addressing the basic needs and care of their infants, with fathers adopting a supportive role [2]. Both the mother and father actively contribute to the baby’s healthy emotional development [3].

With the arrival of a new family member, parents must anticipate and adjust to additional roles and responsibilities [4]. While traditional gender roles often associate fathers primarily with financial matters and mothers with child care [5], the concept of fatherhood has changed. Nowadays, fathers are not only providers but also offer emotional support, dedicate time, express affection, and take responsibility for childcare [6, 7]. The maternal well-being is crucial for child health; nevertheless, the influence of the father on child and family health should not be overlooked [8]. It is asserted that simple interventions facilitating and fostering paternal-infant attachment, such as participating in activities like changing and bathing the baby, can effectively enhance fathers’ engagement in infant care [9]. Research indicates that fathers’ active involvement in infant care positively impacts children’s psychological, cognitive, social, and behavioral development [10, 11]. Fathers may be encouraged to take on childcare responsibilities such as feeding and engaging in skin-to-skin contact from the early stages of infancy [8].

Bowlby posits that a child’s bidirectional social and psychological interaction with a caregiver, aimed at eliciting protective and comforting behaviors, forms the foundation of attachment theory [12]. In attachment theory, the key emphasis lies in the quality of the relationship between the infant and the caregiver. When this relationship evolves positively, encompassing the baby’s sense of safety, fulfillment of basic needs, establishment of emotional connections, and meeting of expectations, secure attachment is formed [13]. Secure attachment, fostered through positive interactions, particularly with the father during infancy, significantly impacts the child’s overall development [14].

Attachment is commonly associated with mothers in the literature [15]. While there is a wealth of studies on mother-infant attachment [16–20], limited research has focused on paternal-infant attachment. A study investigating the impact of the father-infant relationship on infant development revealed that fathers were actively involved in providing affection, care, and play during the initial months of their infants’ lives. Fathers reported feeling a strong connection to their infants from birth, and infants exhibited higher levels of motor, language, and personal/social development [21]. In another study, where fathers provided 10 min of tactile stimulation once a day for five days, it was concluded that infants showed significant stabilization in physiological parameters such as oxygen saturation levels, heart rate, and respiratory rates [22]. A review of 12 studies on fathers highlighted that father-infant skin-to-skin contact had positive effects on various outcomes, including infants’ temperature and pain, bio-physiological markers, behavioral response, parental role acquisition, father interaction behavior, and father stress and anxiety [23]. In a study investigating the impact of attachment training on father-fetal attachment and parental anxiety, Setodeh et al. [24] reported that the training led to an increase in father-fetal attachment and a decrease in parental anxiety. Another study examining fathers’ attachment to their infants, conducted by Aslan et al. [25], revealed that fathers exhibited very high levels of attachment to their infants, with socio-demographic characteristics showing no significant influence on attachment. Consequently, the literature suggests that, akin to mother-infant attachment, paternal-infant attachment confers numerous benefits to the infant.

Sociocultural norms and values pertaining to fathers’ involvement in childcare have changed over time, shaping fathers’ personal norms and behaviors [26]. Exploration of the impact of fathers on their children’s health and development has been on the rise in years [27]. While the responsibility for infant care is universally ascribed to mothers, engaging fathers in the care of their infants has been shown to not only enhance the paternal-infant attachment, contributing to child development, but also yield positive health benefits for the infant, mother, father, and the entire family [28]. It is crucial for mothers to encourage shared caregiving between fathers and infants from the outset, providing fathers with the opportunity to participate actively. The involvement of fathers in the baby’s care and daily activities is believed to strengthen the attachment between father and infant. This study, conducted with fathers of infants aged 6–12 months, addresses the lack of quantitative evidence on the relationship between father involvement in infant care and paternal-infant attachment in the Türkiye context. Hence, the aim of this study is to ascertain the relationship between fathers’ engagement in infant care and their levels of attachment to their infants.

### Research questions

- Do fathers’ involvement in infant care levels vary according to sociodemographic factors?
- Do fathers’ infant attachment levels vary according to sociodemographic factors?
- Does a relationship exist between fathers’ involvement in infant care levels and paternal-infant attachment?
- Does fathers’ involvement in infant care have a significant effect on paternal-infant attachment?

## Methods

### Study design

This research is descriptive-cross-sectional and correlational in design. The study was conducted in 6 Family Health Centers in a provincial center in southeastern Türkiye. Data were collected between February and December 2022.

### Population and sample

The study’s population comprised fathers who had applied to Family Health Centers for their infants’ vaccinations. The study was concluded with the participation of 255 fathers who willingly joined, exhibited no communication hindrances, and had no vision-related issues. An a priori power analysis was conducted using the G*Power 3.1 software to determine the required sample size prior to data collection. The analysis was based on the statistical test “F tests – Linear multiple regression: Fixed model, R^2^ deviation from zero.” A medium effect size (f^2^ = 0.15; small = 0.02, medium = 0.15, large = 0.35, according to Cohen), a significance level of α = 0.05, and a statistical power of 1 − β = 0.95 were specified. According to the model, which included five independent variables (age, educational status, occupation, economic status, and age at marriage), the minimum required sample size was calculated to be 138 participants. In this study, data from 255 fathers were analyzed, exceeding the required minimum and ensuring adequate statistical power for the analyses.

### Data collection tools

The data of the study were collected by using “Descriptive Information Form”, “Fathers’ Involvement in Infant Care Questionnaire” and “Paternal-Infant Attachment Scale”.

#### Descriptive information form

It is a form prepared by the researchers that covers the sociodemographic characteristics of the fathers and consists of 13 questions.

#### Fathers’ involvement in infant care questionnaire (FIICQ)

The Fathers’ Involvement in Infant Care Questionnaire, developed by Kuruçırak and Kulakaç [29], is a five-point Likert-type instrument designed to evaluate the extent of paternal involvement in infant care. Responses range from 1 (Never) to 5 (Always), with higher scores indicating greater engagement in caregiving activities. The questionnaire consists of three subscales: (1) Physical Care (13 items), (2) Personal Development and Health (8 items), and (3) Allocating Time and Play Activities (7 items).

The original validation study reported strong internal consistency, with Cronbach’s alpha coefficients of 0.95 for the overall scale and 0.93, 0.89, and 0.86 for the respective subscales [29]. In the present study, internal consistency analyses confirmed the robustness of the scale, yielding Cronbach’s alpha values of 0.93 (total scale), 0.87 (Physical Care), 0.82 (Personal Development and Health), and 0.94 (Allocating Time and Play Activities). These findings reaffirm the scale’s reliability in assessing paternal engagement in diverse caregiving contexts.

#### Paternal-infant attachment scale (P-IAS)

The Paternal-Infant Attachment Scale, originally developed by Condon et al. [30], is a validated instrument designed to assess the attachment between fathers and their infants during the postnatal period. The scale was adapted for the Turkish population by Güleç and Kavlak [3], who conducted an extensive validity and reliability study. The adaptation process preserved the original three-factor structure while refining the scale to 18 items, slightly modifying the item composition to enhance conceptual clarity and measurement precision. The scale comprises three interrelated subdimensions that capture distinct yet complementary aspects of paternal attachment. The Patience and Tolerance dimension assesses a father’s capacity for patience and attentiveness in responding to the infant’s needs, reflecting his ability to provide consistent care. The Pleasure in Interaction subdimension measures the extent to which fathers actively participate in interactions with their infants, emphasizing moments of shared pleasure and responsiveness. The Affection and Pride factor evaluates the emotional depth of paternal love and the intrinsic sense of closeness fathers experience with their infants.

Each item is rated on a five-point Likert scale ranging from 1 (Strongly Disagree) to 5 (Strongly Agree), with 12 reverse-scored items (4, 5, 7, 8, 9, 10, 11, 12, 13, 14, 15, 16) adjusted accordingly to maintain the directional integrity of the scale. Higher total scores indicate greater levels of paternal-infant attachment, with possible scores ranging from 18 to 95. The psychometric properties of the scale have been well established, with Cronbach’s alpha coefficients reported as 0.81 at six months postpartum and 0.78 at twelve months postpartum in the original study by Condon et al. [30], indicating high internal consistency. The Turkish adaptation by Güleç and Kavlak [3] demonstrated comparable reliability indices, confirming the instrument’s robustness in the adapted context. In the present study, the Cronbach’s alpha coefficient was calculated as 0.85, further affirming the scale’s reliability in measuring paternal-infant attachment.

### Data collection

After obtaining informed consent from the fathers who applied to the family health center to have their babies vaccinated, data were collected by face-to-face interview technique. Data was collected in a quiet environment in the waiting room of the family health center. The questionnaire response time for each participant was approximately 15–20 min.

### Data analysis

All statistical analyses were conducted using IBM SPSS Statistics version 23. The data were evaluated, employing number, percentage, mean, and standard deviation statistics to describe the characteristics of the fathers. Kurtosis and Skewness values were examined to ascertain the normal distribution of the research variables. Furthermore, the Kolmogorov–Smirnov test was utilized to assess the suitability of the data for normal distribution, confirming that the variables exhibited normal distribution. Parametric methods were employed for data analysis, including T-test and one-way analysis of variance (ANOVA) to compare descriptive characteristics, total scores, and subscale scores on the scale. Following significant ANOVA results, Tukey’s Honestly Significant Difference (HSD) post hoc test was performed to determine specific group differences. Pearson correlation and linear regression analyses were conducted. Additionally, Cronbach’s alpha coefficient was calculated to evaluate the scale’s reliability. The results were assessed at a 95% confidence interval, with significance set at p < 0.05.

### Ethical considerations

Before starting the research, Ethics Committee Approval was obtained for the study from Gaziantep University Clinical Research Ethics Committee (Meeting Date: 1 December 2021, Decision No: 2021/384). Subsequently, permission was granted to carry out the research within Family Health Centers affiliated with the Provincial Health Directorate (Document ID: 28 January 2022/90). Additionally, permission was obtained from the relevant authors for the utilization of the scales employed in the study. Individuals participating in the research were duly informed about the study’s objectives and procedures, and their informed consent was obtained, adhering to ethical principles. Written informed consent was obtained from all the fathers included in the study. The study strictly adhered to the principles outlined in the Helsinki Declaration of Human Rights.

## Results

The study examined various sociodemographic and contextual factors influencing fathers’ involvement in infant care and their attachment levels, as measured by the FIICQ and P-IAS. A total of 255 fathers participated, 57.3% having infants aged 6–8 months. The results showed no significant differences in paternal involvement or attachment based on the infant’s age, indicating that engagement levels remained consistent across early infancy (p > 0.05). However, fathers aged 30 years or younger demonstrated significantly higher overall attachment scores (t = 2.214, p = 0.028) and greater in the Pleasure in Interaction dimension (t = 1.991, p = 0.048), suggesting that younger fathers may be more emotionally invested in bonding activities with their infants. Educational attainment emerged as a strong determinant of paternal involvement, with university-educated fathers exhibiting the highest FIICQ and P-IAS scores across all subscales (p < 0.001). Post hoc analyses confirmed that fathers with elementary education reported significantly lower involvement compared to those with secondary education or higher. Similarly, fathers employed as civil servants reported the highest engagement levels in infant care (F = 8.226, p < 0.001), while laborers demonstrated the lowest scores across most subscales. Economic status was another key predictor, with fathers from middle-income households scoring significantly higher on overall paternal involvement (t = −3.134, p = 0.002), Physical Care (t = −4.238, p < 0.001), and attachment scores (t = −2.671, p = 0.008), indicating that financial stability may facilitate greater father-child interaction. Additionally, none of the participants reported a high-income status (Table 1).

**Table 1:** Comparison of the fathers’ FIICQ and P-IAS mean scores according to descriptive characteristics (n = 255)

**Characteristics**	**n (%)**	**FIICQ** **Mean** ± **SD**	**Physical Care Mean** ± **SD**	**Personal Development and Health** **Mean** ± **SD**	**Allocating Time and Play Activities** **Mean** ± **SD**	**P-IAS** **Mean** ± **SD**	**Patience and Tolerance** **Mean** ± **SD**	**Pleasure in Interaction** **Mean** ± **SD**	**Affection and Pride** **Mean** ± **SD**
**Baby’s age**									
6–8 months	146 (57.3)	84.41 ± 15.41	31.86 ± 7.74	26.83 ± 5.09	25.71 ± 4.35	72.85 ± 8.19	33.97 ± 4.08	25.73 ± 4.08	13.13 ± 1.78
9–12 months	109 (42.7)	84.19 ± 16.86	32.07 ± 7.95	26.33 ± 5.47	25.77 ± 4.81	71.19 ± 9.94	32.95 ± 4.58	25.32 ± 4.75	12.91 ± 1.82
t		0.111	−0.205	0.745	−0.117	1.460	1.869	0.744	1.002
p		0.912	0.838	0.457	0.907	0.146	0.063	0.458	0.317
**Father’s age**									
≤ 30 years	136 (53.3)	85.38 ± 16.50	32.52 ± 8.22	26.92 ± 5.34	25.93 ± 4.61	73.30 ± 9.27	34.03 ± 4.54	26.06 ± 4.48	13.19 ± 1.75
≥ 31 years	119 (46.7)	83.10 ± 15.43	31.31 ± 7.31	26.27 ± 5.14	25.52 ± 4.47	70.82 ± 8.53	32.97 ± 4.00	24.98 ± 4.20	12.86 ± 1.84
t		1.131	1.234	0.984	0.723	2.214	1.960	1.991	1.492
p		0.259	0.218	0.326	0.470	0.028*	0.051	0.048*	0.137
**Father’s educational status**									
Elementary School^a^	21 (8.2)	68.52 ± 20.72	26.80 ± 8.70	20.71 ± 6.51	21.00 ± 6.03	59.88 ± 15.04	28.76 ± 6.87	19.09 ± 6.17	12.03 ± 2.63
Secondary School^b^	32 (12.5)	82.06 ± 14.75	31.65 ± 7.16	25.28 ± 5.29	25.12 ± 4.29	70.67 ± 8.06	33.05 ± 4.52	24.80 ± 3.61	12.81 ± 1.99
High School^c^	147 (57.6)	85.08 ± 13.63	31.48 ± 6.71	27.57 ± 4.48	26.02 ± 3.96	72.79 ± 6.90	33.58 ± 3.49	26.29 ± 3.45	12.90 ± 1.60
University and above^d^	55 (21.6)	89.61 ± 17.04	35.36 ± 9.26	27.10 ± 5.11	27.14 ± 4.40	75.94 ± 7.46	35.53 ± 3.56	26.50 ± 4.17	13.91 ± 1.49
F		10.117	7.191	12.919	10.898	20.678	14.583	22.303	7.439
p		< 0.001*	< 0.001*	< 0.001*	< 0.001*	< 0.001*	< 0.001*	< 0.001*	< 0.001*
Post hoc analysis		b = c = d > a	d > a = b = c	d > b = c > a	b = c = d > a	d > b = c > a	d > b = c > a	b = c = d > a	d > a = b = c
**Father’s occupation**									
Tradesmen/self-employment^a^	61 (23.9)	83.04 ± 16.46	31.68 ± 8.06	25.91 ± 5.53	25.44 ± 4.64	71.05 ± 9.42	33.37 ± 4.38	24.64 ± 4.69	13.03 ± 1.86
Laborer^b^	152 (59.6)	82.37 ± 15.77	30.65 ± 7.34	26.40 ± 5.15	25.32 ± 4.59	71.37 ± 8.94	33.23 ± 4.34	25.30 ± 4.25	12.83 ± 1.76
Civil Servant^c^	42 (16.5)	93.21 ± 13.38	37.07 ± 7.21	28.45 ± 4.88	27.69 ± 3.73	76.50 ± 7.36	34.90 ± 4.00	27.79 ± 3.65	13.80 ± 1.69
F		8.226	12.098	3.288	4.778	6.145	2.528	7.387	4.932
p		< 0.001*	< 0.001*	0.039*	0.009*	0.002*	0.082	0.001*	0.008*
Post hoc analysis		c > a = b	c > a = b	c > a = b	c > a = b	c > a = b	-	c > a = b	c = a > b
**Economics status**									
Low	133 (52.2)	81.36 ± 14.25	30.00 ± 6.35	26.26 ± 4.94	25.09 ± 4.28	70.71 ± 8.57	32.93 ± 4.18	25.14 ± 4.39	12.64 ± 1.65
Middle	122 (47.8)	87.54 ± 17.23	34.08 ± 8.69	27.01 ± 5.56	26.45 ± 4.73	73.69 ± 9.24	34.20 ± 4.40	26.01 ± 4.34	13.47 ± 1.86
t		−3.134	−4.238	−1.144	−2.410	−2.671	−2.356	−1.597	−3.807
p		0.002*	< 0.001*	0.254	0.017*	0.008*	0.019*	0.112	< 0.001*
**Spouse occupation**									
Housewife^a^	214 (83.9)	83.23 ± 16.36	31.24 ± 7.72	26.46 ± 5.43	25.52 ± 4.70	71.48 ± 9.17	33.29 ± 4.43	25.27 ± 4.49	12.91 ± 1.82
Laborer^b^	15 (5.9)	82.46 ± 16.15	31.73 ± 8.71	25.80 ± 4.26	24.93 ± 3.78	74.90 ± 7.79	35.25 ± 3.24	26.58 ± 3.72	13.06 ± 1.72
Civil Servant^c^	26 (10.2)	94.34 ± 7.98	37.96 ± 5.39	28.42 ± 3.80	27.96 ± 2.69	75.95 ± 7.07	34.60 ± 3.64	27.30 ± 3.30	14.04 ± 1.31
F		5.902	9.100	1.821	3.649	3.664	2.335	2.951	4.661
p		0.003*	< 0.001*	0.164	0.027*	0.027*	0.099	0.054	0.010*
Post hoc analysis		c > a = b	c > a = b	-	c > a = b	**-**	-	-	c > a = b
**Spouse’s Education Status**									
Elementary School^a^	44 (17.3)	71.72 ± 17.08	27.36 ± 7.31	22.00 ± 5.62	22.36 ± 5.12	64.10 ± 12.71	30.07 ± 6.09	21.90 ± 5.71	12.13 ± 2.24
Secondary School^b^	65 (25.5)	83.92 ± 12.75	31.64 ± 6.65	26.96 ± 4.08	25.30 ± 3.67	72.75 ± 6.24	33.83 ± 3.15	26.11 ± 3.36	12.79 ± 1.74
High School^c^	103 (40.4)	86.06 ± 12.59	31.51 ± 6.50	27.86 ± 4.22	26.68 ± 3.60	73.58 ± 6.89	34.27 ± 3.44	26.17 ± 3.34	13.13 ± 1.56
University and above^d^	43 (16.9)	93.62 ± 18.91	38.18 ± 9.10	27.86 ± 6.14	27.58 ± 5.28	76.00 ± 7.85	34.90 ± 3.90	27.00 ± 4.54	14.10 ± 1.32
F		17.031	16.981	16.802	14.005	18.438	13.724	14.903	10.167
p		< 0.001*	< 0.001*	< 0.001*	< 0.001*	< 0.001*	< 0.001*	< 0.001*	< 0.001*
Post hoc analysis		d > b = c > a	d > b = c > a	d = b = c > a	d > b = c > a	d = b = c > a	d = b = c > a	d = b = c > a	d > c = b > a
**Marriage year**									
< 10 years	171 (67.1)	86.55 ± 16.14	33.23 ± 8.16	27.14 ± 5.15	26.17 ± 4.46	73.46 ± 8.59	34.00 ± 4.19	26.18 ± 4.14	13.27 ± 1.71
≥ 10 years	84 (32.9)	79.77 ± 14.84	29.34 ± 6.35	25.57 ± 5.33	24.85 ± 4.60	69.46 ± 9.26	32.59 ± 4.46	24.29 ± 4.59	12.57 ± 1.88
t		3.236	3.835	2.259	2.193	3.399	2.474	3.290	2.956
p		0.001*	< 0.001*	0.025*	0.029*	0.001*	0.014*	0.001*	0.003*
**Relationship status**									
Good	157 (61.6)	81.39 ± 14.89	30.45 ± 6.70	25.94 ± 5.22	24.98 ± 4.41	69.94 ± 9.01	32.55 ± 4.35	24.83 ± 4.29	12.55 ± 1.75
Very good	98 (38.4)	89.01 ± 16.70	34.35 ± 8.86	27.70 ± 5.13	26.94 ± 4.51	75.66 ± 7.83	35.11 ± 3.80	26.72 ± 4.28	13.81 ± 1.61
t		−3.788	−3.737	−2.625	−3.423	−5.171	−4.795	−3.426	−5.748
p		< 0.001*	< 0.001*	0.009*	0.001*	< 0.001*	< 0.001*	0.001*	< 0.001*
**Baby’s gender**									
Girl	134 (52.5)	86.27 ± 16.74	32.67 ± 8.55	27.37 ± 5.46	26.22 ± 4.57	72.57 ± 8.70	33.68 ± 4.26	25.76 ± 4.27	13.13 ± 1.87
Boy	121 (47.5)	82.15 ± 14.95	31.15 ± 6.87	25.79 ± 4.89	25.20 ± 4.47	71.66 ± 9.33	33.38 ± 4.40	25.33 ± 4.50	12.94 ± 1.72
t		2.063	1.556	2.420	1.793	0.805	0.546	0.767	0.846
p		0.040*	0.121	0.016*	0.074	0.422	0.585	0.444	0.398
**Type of birth**									
Vaginal section	161 (63.1)	82.00 ± 14.86	30.75 ± 6.96	26.07 ± 5.10	25.18 ± 4.30	71.82 ± 10.00	33.34 ± 4.80	25.55 ± 4.61	12.93 ± 1.86
Caesarian section	94 (36.9)	88.28 ± 17.15	34.02 ± 8.76	27.56 ± 5.39	26.70 ± 4.80	72.68 ± 6.98	33.88 ± 3.35	25.57 ± 3.96	13.22 ± 1.68
t		−3.070	−3.281	−2.200	−2.609	−0.805	−1.060	−0.040	−1.258
p		0.002*	0.001*	0.029*	0.010*	0.421	0.290	0.968	0.210
**Number of children**									
1^a^	79 (31.0)	89.86 ± 18.56	35.10 ± 9.64	27.84 ± 5.43	26.91 ± 4.86	75.69 ± 7.87	34.78 ± 3.70	27.09 ± 4.21	13.81 ± 1.49
2^b^	84 (32.9)	84.36 ± 10.86	31.71 ± 5.80	26.96 ± 3.91	25.69 ± 3.53	72.80 ± 7.15	33.90 ± 4.03	25.92 ± 3.17	12.97 ± 1.49
3^c^	57 (22.4)	82.15 ± 16.89	30.78 ± 7.03	25.96 ± 6.01	25.40 ± 5.04	68.67 ± 10.24	32.20 ± 4.31	23.87 ± 5.21	12.59 ± 2.00
≥ 4^d^	35 (13.7)	75.22 ± 14.32	27.34 ± 5.70	24.11 ± 5.52	23.77 ± 4.49	68.20 ± 9.95	32.05 ± 5.34	23.96 ± 4.47	12.18 ± 2.15
F		7.838	9.656	4.705	4.198	10.312	5.878	8.444	9.602
p		< 0.001*	< 0.001*	0.003*	0.006*	< 0.001*	0.001*	< 0.001*	< 0.001*
Post hoc analysis		a > b = c > d	a > b = c > d	a = b > c = d	a = b = c > d	a > b = c = d	a = b > c > d	a = b > c = d	a > b = c = d
**Time spent with baby**									
0–2 hours^a^	56 (22.0)	77.16 ± 17.65	29.42 ± 7.52	24.28 ± 6.12	23.44 ± 5.01	65.89 ± 11.72	30.52 ± 5.44	23.32 ± 5.55	12.04 ± 1.99
3–5 hours^b^	145 (56.8)	86.86 ± 15.56	32.93 ± 8.02	27.58 ± 4.96	26.34 ± 4.43	73.92 ± 7.38	34.17 ± 3.57	26.44 ± 3.76	13.30 ± 1.69
6 h and more^c^	54 (21.2)	84.92 ± 13.29	31.96 ± 7.08	26.46 ± 4.26	26.50 ± 3.50	73.86 ± 6.72	34.96 ± 3.39	25.52 ± 3.75	13.37 ± 1.50
F		7.857	4.148	8.473	9.805	19.849	20.898	11.035	11.989
p		< 0.001*	0.017*	< 0.001*	< 0.001*	< 0.001*	< 0.001*	< 0.001*	< 0.001*
Post hoc analysis		b = c > a	b = c > a	b = c > a	b = c > a	b = c > a	b = c > a	b = c > a	b = c > a

FIICQ: Fathers’ Involvement in Infant Care Questionnaire; P-IAS: Paternal-Infant Attachment Scale; SD: Standard Deviation; t: Independent Samples T-Test; F: One-way analysis of variance (ANOVA)Superscript letters (^a, b, c, d^) indicate significant differences between groups according to Tukey’s HSD post-hoc test (p < 0.05). Groups that do not share the same letter differ significantly; *p < 0.05

Other familial and relational factors also played a significant role in determining fathers’ engagement in infant care. Fathers with working spouses—particularly those whose partners were civil servants—reported significantly higher involvement in caregiving activities (F = 5.902, p = 0.003) and attachment levels (F = 3.664, p = 0.027), suggesting that paternal participation increases when caregiving responsibilities are more equitably distributed. Similarly, fathers whose spouses had a university education exhibited the highest FIICQ and P-IAS scores across all dimensions (p < 0.001), reinforcing the link between parental education and paternal engagement. Relationship quality also influenced involvement, as fathers who rated their marital relationship as “very good” demonstrated significantly greater engagement across all FIICQ subscales (p < 0.001) and higher attachment scores (p < 0.001). The number of children in the household inversely correlated with paternal involvement, with first-time fathers scoring significantly higher on all FIICQ and P-IAS subscales compared to fathers with multiple children (p < 0.001), suggesting that paternal engagement diminishes as family size increases. Time spent with the infant was a key determinant of involvement, with fathers who spent three or more hours daily with their child reporting significantly higher scores across all subscales of both FIICQ and P-IAS (p < 0.001). These findings underscore the crucial role of time investment in fostering both caregiving behaviors and paternal-infant attachment (Table 1).

The correlation analysis presented in Table 2 highlights several significant associations between fathers’ involvement in infant care (FIICQ) and paternal-infant attachment (P-IAS), along with their respective subdimensions. A strong positive correlation was observed between overall FIICQ and P-IAS scores (r = 0.607, p < 0.001), suggesting that greater paternal involvement in infant care is associated with stronger paternal-infant attachment. Among the subdimensions, Allocating Time and Play Activities demonstrated the highest correlation with P-IAS (r = 0.616, p < 0.001), followed closely by Personal Development and Health (r = 0.570, p < 0.001) and Physical Care (r = 0.501, p < 0.001). These findings indicate that fathers who engage more in shared activities and developmental caregiving report higher levels of emotional connection with their infants. Further examination of the P-IAS subdimensions revealed that Pleasure in Interaction exhibited the strongest correlation with FIICQ (r = 0.623, p < 0.001), particularly with Allocating Time and Play Activities (r = 0.622, p < 0.001) and Personal Development and Health (r = 0.615, p < 0.001). This suggests that fathers who actively engage in interactive and developmental caregiving activities experience greater shared enjoyment and bonding with their infants. Additionally, Affection and Pride displayed moderate yet significant correlations with FIICQ and its subdimensions, particularly Allocating Time and Play Activities (r = 0.497, p < 0.001), reinforcing the importance of interactive caregiving in fostering paternal attachment.

**Table 2:** The relationship between fathers’ FIICQ and P-IAS scores

**Scales and Sub-dimensions**	**P-IAS Total Score**	Patience and Tolerance	Pleasure in Interaction	Affection and Pride	**FIICQ** **Total Score**	Physical Care	Personal Development and Health	Allocating Time and Play Activities
**P-IAS Total Score**	r = 1							
Patience and Tolerance	r = 0.879**	r = 1						
Pleasure in Interaction	r = 0.883**	r = 0.590**	r = 1					
Affection and Pride	r = 0.741**	r = 0.555**	r = 0.563**	r = 1				
**FIICQ Total Score**	r = 0.607**	r = 0.434**	r = 0.623**	r = 0.477**	r = 1			
Physical Care	r = 0.501**	r = 0.360**	r = 0.501**	r = 0.422**	r = 0.914**	r = 1		
Personal Development and Health	r = 0.570**	r = 0.400**	r = 0.615**	r = 0.395**	r = 0.897**	r = 0.674**	r = 1	
Allocating Time and Play Activities	r = 0.616**	r = 0.446**	r = 0.622**	r = 0.497**	r = 0.914**	r = 0.722**	r = 0.847**	r = 1

r: Correlation coefficient; FIICQ: Fathers’ Involvement in Infant Care Questionnaire; P-IAS: Paternal-Infant Attachment Scale; **Correlation is significant at the 0.01 level (2-tailed)

Table 3 presents the results of a linear regression analysis examining the predictive relationship between fathers’ involvement in infant care (FIICQ) and paternal-infant attachment (P-IAS). The model explains 36.8% of the variance in paternal-infant attachment (R^2^ = 0.368, p < 0.001), indicating a substantial association between paternal caregiving engagement and attachment outcomes. The regression coefficient for FIICQ total score (B = 0.341, β = 0.607, p < 0.001) demonstrates a statistically significant positive association, suggesting that as fathers’ involvement in infant care increases, their attachment to their infants strengthens. The standardized coefficient (β = 0.607) reflects a moderately strong effect, highlighting the importance of paternal participation in caregiving for fostering attachment. The confidence interval for the predictor variable (95% CI: 0.285–0.397) confirms the precision of the estimate, reinforcing the robustness of this relationship. The model’s F-statistic (F = 147.35, p < 0.001) further validates the overall significance of the regression, underscoring the critical role of active paternal caregiving in enhancing paternal-infant attachment.

**Table 3:** The contribution of participants’ FIICQ total score to P-IAS total score

Predictor Variable	Unstandardized Coefficients (B)	Standard Error (SE)	Standardized Coefficients (β)	t	p-value	95% CI for Difference (LLCI—ULCI)
**Constant**	43.39	2.41	—	17.99	< 0.001	38.64–48.14
**FIICQ Total Score → P-IAS Total Score**	0.341	0.028	0.607	12.14	< 0.001	0.285–0.397
**Model Statistics:** R^2^ = 0.368, Adjusted R^2^ = 0.364, F = 147.35, p < 0.001						

CI = Confidence Interval for Difference; β = Standardized Coefficient; SE = Standard Error; LLCI = Lower Limit of Confidence Interval; ULCI = Upper Limit of Confidence Interval; FIICQ: Fathers’ Involvement in Infant Care Questionnaire; P-IAS: Paternal-Infant Attachment Scale; p < 0.001, indicating statistical significance at the highest level

## Discussion

This study explored the relationship between paternal-infant attachment and paternal involvement in infant care, contributing to a relatively underexplored area in attachment research. While much of the existing literature focuses on maternal-infant attachment, research on paternal attachment remains limited. Previous studies suggest that paternal attachment strengthens through increased interaction with the infant, yet comprehensive guidelines on this process are lacking [31]. This study provides further insight into how fathers’ engagement in caregiving influences their attachment to their infants.

Findings revealed that fathers under the age of 30 demonstrated significantly higher paternal-infant attachment scores, particularly in the enjoyment in interaction subdimension. Additionally, higher paternal education levels were associated with greater involvement in infant care and stronger attachment, both of which were statistically significant (Table 1). However, the literature presents mixed findings on these associations. Schaber et al. [32] found no correlation between paternal age, education, and attachment, while Hall et al. [33] reported an increase in attachment difficulties among more highly educated fathers. Similarly, Taubenheim [34] did not identify a significant relationship between paternal age and attachment. In contrast, studies by Gracia [35] and Gracia & Ghysels [36] indicated that fathers with higher education levels tend to spend more time caring for their young children, though this trend varies across cultural contexts. These discrepancies may stem from sociocultural differences in paternal roles and caregiving expectations. The present findings suggest that younger fathers, as they navigate the new experience of fatherhood, may form stronger attachment with their infants. Furthermore, higher education levels likely contribute to a more conscious and engaged approach to infant care, facilitating increased interaction and, consequently, stronger paternal-infant attachment.

The study further revealed that fathers with moderate economic status exhibited significantly higher scores in both the total and subdimensions of paternal involvement in infant care, as well as in paternal-infant attachment, compared to those with lower economic status (Table 1). This finding aligns with previous research, such as that by Cerezo et al. [37], which reported a higher likelihood of insecure attachment among infants raised in families with low socioeconomic status. Additionally, Raley et al. [38] suggested that fathers dedicate less time to their children as their paid working hours increase, a trend corroborated by our study. In lower socioeconomic environments, parental involvement in infant care is expected to be limited due to increased financial and work-related constraints, leading to reduced interactions and potentially weaker attachment. In contrast, families with greater economic resources likely experience fewer work-related pressures, allowing fathers more opportunities to engage in caregiving and foster stronger attachment with their infants.

Fathers who had been married for less than ten years and those who reported a very good marital relationship demonstrated significantly higher levels of participation in infant care and stronger paternal-infant attachment (Table 1). Although research specifically examining the influence of marriage duration and relationship quality on paternal attachment is scarce, Karakaş and Dağlı [39] emphasized that family dynamics and parental relationships are critical in shaping secure attachment. Their study suggested that marital conflicts negatively impact parent-infant attachment security. The present findings suggest that newly married fathers, particularly those with fewer children, may exhibit heightened enthusiasm and attentiveness toward infant care. Furthermore, fathers in positive marital relationships may be more inclined to engage actively in caregiving, potentially influenced by the desire to foster a harmonious family environment.

The study also indicated that fathers with daughters reported higher levels of involvement in infant care; however, no statistically significant relationship was found between the child’s gender and paternal-infant attachment (Table 1). The literature provides limited clarification on this aspect. While Smyth and Russell [40] observed a diminished father-daughter relationship by the time daughters reached the age of five, their findings do not directly apply to infants, highlighting the importance of developmental differences when interpreting these relationships. Given the cultural perception that fathers may be more attuned to their daughters, greater paternal involvement in infant care might be expected. However, the results suggest that gender does not play a defining role in paternal-infant attachment. This aligns with the findings of Yoshida et al. [41], who examined paternal attachment in Japanese families and reported no significant association between attachment levels and the child’s gender.

The findings indicate that fathers with fewer children demonstrated significantly higher scores in both infant care participation and paternal-infant attachment (Table 1). This suggests that having fewer children allows fathers to dedicate more time and attention to each child, fostering greater interaction. Feldman [42] emphasized that active paternal involvement enhances family dynamics and strengthens parent–child attachment. Similarly, Downey [43] argued that as the number of children increases, parental time and financial resources become more divided, potentially reducing direct father-infant interactions. Thus, fathers in smaller families may engage more deeply in caregiving, leading to stronger attachment relationships.

A significant positive correlation was observed between paternal-infant attachment scores and paternal involvement in infant care (r = 0.607, p = 0.000) (Table 2). Additionally, the regression coefficient for FIICQ total score (B = 0.341, β = 0.607, p < 0.001) demonstrates a statistically significant positive association, suggesting that as fathers’ involvement in infant care increases, their attachment to their infants strengthens (Table 3). Feldman [42] noted that while maternal attachment is largely biologically driven, paternal-infant attachment is shaped by caregiving behaviors, social norms, and personal attitudes. Fathers often cite limited time, lack of experience, and feelings of being secondary to maternal roles—such as breastfeeding—as barriers to involvement [44]. However, increased engagement in caregiving strengthens attachment bonds, fostering a sense of security in infants. This attachment allows infants to trust their primary caregivers, using them as both a secure base for exploration and a source of comfort in times of distress [45].

## Limitations and strengths

This study offers valuable insights into the relationship between paternal-infant attachment and paternal involvement in infant care; however, it has several limitations. First, the cross-sectional design prevents the establishment of causality, limiting the ability to determine whether increased paternal involvement enhances attachment or if fathers with stronger attachment tendencies naturally engage more in infant care. Additionally, self-reported measures may introduce social desirability bias, as fathers might overestimate their caregiving participation. The study was conducted within a specific cultural context, potentially restricting the generalizability of findings to other sociocultural settings where paternal roles and caregiving expectations may differ. Despite these limitations, the study has notable strengths. The use of validated, psychometrically robust scales enhances the reliability of the findings, and the large sample size allows for meaningful statistical comparisons across diverse sociodemographic factors. Furthermore, by addressing an underexplored aspect of early caregiving dynamics, the study contributes to a growing body of literature that underscores the significance of fathers in early childhood development. Future longitudinal research could further clarify the directionality of these associations and examine how paternal involvement changes over time in relation to attachment formation.

## Conclusion

This study underscores the significant relationship between paternal involvement in infant care and paternal-infant attachment, demonstrating that increased engagement in caregiving activities fosters stronger emotional bonds. Key factors such as age, education, economic status, marital relationship quality, and the number of children significantly influence both paternal involvement and attachment, reinforcing the complex interplay of personal, familial, and socio-economic variables. The findings contribute to a growing body of literature emphasizing the evolving role of fathers in early childhood development, challenging traditional caregiving norms and advocating for more inclusive parenting practices. While this study offers valuable insights, cultural variations and external factors such as work-life balance and societal expectations must be further explored to provide a more comprehensive understanding of paternal attachment. Future research should integrate longitudinal methodologies and intervention-based approaches to assess the long-term impact of paternal involvement on child development outcomes. Strengthening paternal-infant attachment through targeted education, policy reforms, and supportive healthcare practices will ultimately enhance family well-being and promote healthier developmental trajectories for children.

## Relevance for clinical practice

The findings of this study emphasize the critical role of paternal involvement in fostering secure paternal-infant attachment, highlighting the need for healthcare professionals, particularly midwives and neonatal nurses, to actively promote father-inclusive caregiving practices. Prenatal and postnatal education programs should integrate structured interventions that encourage fathers to participate in infant care from the earliest stages, reinforcing the importance of their role beyond traditional maternal-centered models. Healthcare settings should adopt father-friendly policies, such as flexible visitation hours in neonatal units and targeted counseling sessions that address paternal concerns, including confidence in caregiving, time constraints, and perceived societal expectations. Additionally, policymakers should advocate for extended and more equitable parental leave policies, allowing fathers to establish stronger bonds with their infants without the pressure of immediate workforce reintegration. By fostering a caregiving culture that supports and normalizes paternal engagement, nursing professionals can contribute to enhanced paternal-infant attachment, ultimately benefiting infant development and family well-being. Future research should explore intervention-based approaches to facilitate paternal involvement, particularly in diverse cultural contexts where traditional gender roles may present barriers to active caregiving participation.

## Data Availability

The data that support the findings of this study are available on request from the corresponding author. The data are not publicly available due to privacy or ethical restrictions.
